# The Effects of rGO Content and Drying Method on the Textural, Mechanical, and Thermal Properties of rGO/Polymer Composites

**DOI:** 10.3390/polym15051287

**Published:** 2023-03-03

**Authors:** Jelena D. Jovanovic, Stevan N. Blagojevic, Borivoj K. Adnadjevic

**Affiliations:** 1 Institute of General and Physical Chemistry, Studentski Trg 12-16/V, 11158 Belgrade, Serbia; 2 Faculty of Physical Chemistry, University of Belgrade, Studentski Trg 12-16, 11158 Belgrade, Serbia

**Keywords:** aerogel, composite, rGO, ambient pressure drying, freeze-drying, properties

## Abstract

Composite hydrogels samples consisting of poly(methyl methacrylate/butyl acrylate/2-hydroxyethylmethacrylate) (poly-OH) and up to 60% reduced graphene oxide (rGO) containing rGO were synthesized. The method of coupled thermally induced self-assembly of graphene oxide (GO) platelets within a polymer matrix and in situ chemical reduction of GO was applied. The synthesized hydrogels were dried using the ambient pressure drying (APD) and freeze-drying (FD) methods. The effects of the weight fraction of rGO in the composites and the drying method on the textural, morphological, thermal, and rheological properties were examined for the dried samples. The obtained results indicate that APD leads to the formation of non-porous xerogels (X) of high bulk density (D), while FD results in the formation of highly porous aerogels (A) with low D. An increase in the weight fraction of rGO in the composite xerogels leads to an increase in D, specific surface area (SA), pore volume (Vp), average pore diameter (dp), and porosity (P). With an increase in the weight fraction of rGO in A-composites, the D values increase while the values of SP, Vp, dp, and P decrease. Thermo-degradation (TD) of both X and A composites takes place through three distinct steps: dehydration, decomposition of residual oxygen functional group, and polymer chain degradation. The thermal stabilities (TS) of the X-composites and X-rGO are higher than those of the A-composites and A-rGO. The values of the storage modulus (E’) and the loss modulus (E”) of the A-composites increase with the increase in their weight fraction of rGO.

## 1. Introduction

Aerogels are three-dimensional (3D) porous materials that are the lightest solid materials known, presenting a novel class of materials. Aerogels exhibit some unique properties such as low bulk density (D = 3 kg/m^3^), high porosity (P = 90–99%), large specific surface areas (SA), low thermal conductivity (k = 0.014 W/mK at room temperature), low refractive index, and low dielectric constants [1]. The most important class of carbon aerogels are graphene-based aerogels (GAs), which are characterized by low D = 9–96 mg/cm^3^, and high pore volume (V_p_ = 1.5–2.9 cm^3^/g), SA = 130–1800 m^2^/g, specific capacitance, and cyclic stability. GAs are promising materials that attract significant attention and have a number of possible applications such as electrodes materials [2,3], supercapacitors [4], gas storage and separation [5], sensors [6] catalyst supports [7,8], photonic band gap materials [9], separation membranes [10], phase- changing materials [11], etc.

In the literature, numerous methods have been described for producing GAs that can be classified into several categories: hydrothermal reduction [12], chemical reduction [13], cross-linking [14], template-mediated assembly [15,16], chemical vapor deposition (CVD) [17], 3D printing [18], and 3D printing-freeze drying [1]. Comprehensive literature overviews in the field of polymer-graphene-based composites, in regard to their, preparation, characterization, and applications are given in the papers of M. Zhang et al. [19], A. Arzac et al. [20], W. C. Hu et al. [21], C. N. Nwosu et al., [22], Y. Fadil et al. [23].

Methods of reactive emulsion mixing have been described for the synthesis of poly(methyl methacrylate/butyl acrylate/2-hydroxyethylmethacrylate)/rGO composite (PMMA/BA/HEMA-/rGO) [24] and in situ synthesis by semicontinuous emulsion polymerization in the presence of polyurethane (PU) [25]. Composite hydrogel supports by self-assembly of GO nanoplatelets in a polymer latex matrix with the addition of ascorbic acid (AAs) as a GO reducing agent have been synthesized by using methyl methacrylate (MMA) and butyl acrylate (BA) copolymer latexes, functionalized by Br and OH. [26]. Poly (methyl methacrylate-co- butyl acrylate-co-2-hydroxyethyl methacrylate) (PMMA/BA/HEMA) filled with graphene nanoribbons (GNRs) reinforced nanocomposites have been synthesized and their sensing properties investigated [27].

Zhang et al. synthesized composites of polyacrylates with different functional groups: carboxyl P(MMA/BA/AA), hydroxyl P(MMA/BA/HEA), and acylamino groups P(MMA/BA/AM) and amino-functionalized GO via emulsion polymerization [28]. Advancements in the synthesis of graphene-based polymeric nanocomposites have been presented [29]. M. D. Prasad et al., presented synthesis methods for graphene nanoribbons (GNRs) reinforced polymer nanocomposites along with rheological and thermal properties [30]. A simple method for obtaining colloidally stable graphene-based polymer nanocomposite has been developed [31]. 

The drying method for GA preparation is an important factor that limits their large-scale production. In the literature, numerous drying methods have been described for the preparation of 3D porous GA: supercritical drying (SC), [32], freeze-drying [33], vacuum drying [34], ambient pressure drying [35], and a combination of freeze-thawing and APD [36]. APD is found to be more suitable for the large-scale production of GA. However, during APD drying, strong capillary forces act in the initial structure of the partially dried hydrogel in the small pores, which leads to the collapse of the porous structure and prevents the formation of an aerogel [35]. For that reason, the most frequently applied drying methods used to obtain GA are mainly the two drying methods: SC and FD. Advances in 3D-GA preparation by different drying methods have been reviewed [37]. Zhang et al. investigated the effects of SC and FD methods on the GAs properties. [38].

GAs with enhanced mechanical, thermal, and electrical properties have been prepared by supercritical ethanol drying and high-temperature reduction [39]. X. Jing et al. synthesized GAs samples by the sol-gel method, which have been dried by SD and FD methods. The drying method notably influenced GA microstructure and mechanical compression [40]. Wang et al. investigated the effect of drying conditions on structure and electrochemical performance using N-doped graphene as a model material. When the graphene hydrogel was directly freeze-dried, an interconnected porous structure can be obtained, whereas, heat-dried and pre-frozen samples had layered structures [41]. A novel method based on APD for the preparation of GA with superelasticity and multifunctionality has been presented. The method was based on recasting the partially reduced GO hydrogel with the ice-template method [36]. 

To the best of our knowledge, literature data concerning the effects of weight fraction of rGO and method of drying on the textural, morphological, thermal, and rheological properties of composite hydrogels (CHG) are very sparse. The aim of this work was to investigate the effects of weight fraction of rGO and method of drying (APD and FD) on texture properties: (specific surface area (SA), pores volume (V_p_), average pore diameter (d_p_), porosity (P) and bulk density (D)), along with their morphology, thermal stability, and rheological properties (storage modulus (E’) and loss modulus (E”)) of aerogels (A) and xerogels (X), obtained by the method of coupled thermally induced self-assembly of graphene oxide (GO) platelets within a polymer matrix and in situ chemical reduction of GO.

## 2. Materials and Methods

### 2.1. Materials

Technical grade methyl methacrylate (MMA), and butyl acrylate (BA), Quimidroga, Barcelona, Spain, 2-hydroxy ethyl methacrylate (HEMA), Sigma-Aldrich Chemie, Gmbh, Steinheim, Germany, were used as monomers. Potassium persulfate (p.a.) (KPS), and 4,4-azobis(4-cyanovaleric acid) (V-501) (p.a.), Sigma-Aldrich Chemie, Gmbh, Steinheim, Germany were used as initiators. Sodium dodecyl sulfate (SDS) (p.a.), Sigma-Aldrich, Sigma-Aldrich Chemie, Gmbh, Steinheim, Germany and polyvinylpyrrolidone (PVP) (puriss) with an average weight molar weight Mw = 10,000, purchased from Sigma-Aldrich Chemie, Gmbh, Steinheim, Germany, were used as surfactants. Graphene oxide (GO) aqueous dispersion (concentration 4 g/L GO, >95% monolayer content), Graphenea, San Sebastian, Spian, and ascorbic acid (AsA) (p.a.), Acros, Gell, Belgioum. All the chemicals were used without further purification. Oxygen-free-grade nitrogen and double-deionized water were used throughout the experimentation. 

### 2.2. Synthesis of Composite Hydrogels and rGO Hydrogel

The synthesis of composite hydrogel (CHG) was performed by the method of coupled thermally induced self-assembly of GO platelets within a polymer matrix and in situ chemical reduction of GO. An aqueous dispersion of OH functionalized polymer based on MMA/BA/HEMA in weight fractions of 49.5/49.5/1, was prepared by the method of seeded semicontinuous emulsion polymerization of poly-OH. Details about the synthesis and characterization are given elsewhere [26]. The synthesis of CHG was performed by the method of coupled thermally induced self-assembly of graphene oxide (GO) platelets within a polymer matrix and in situ chemical reduction of GO. Briefly, GO dispersion (50 g) was mixed in different weight fractions rGO with poly-OH (1.25/1; 0.63/1, and 0.42/1) for 3 h. The mixtures were afterward heated to 70 °C and AsA reducing agent (AsA/GO weight fraction of 1) was added and left to react for 3 h. In order to remove residual reactants, hydrogels were dialyzed for one week in ultrapure water controlling the conductivity until it remained constant. To that end, a Spectral/Por dialysis membrane (Spectrumlabs) with a molecular cut-off of 12,000–14,000 Da was used. The rGO hydrogel was synthesized by the same procedure as GH without the presence of poly-OH. 

Synthesized poly-OH, CHG, and rGO-HG were dried by two methods: freeze drying (FD) of hydrogels, which was carried out under vacuum in a lyophilizer (Telstar LyoQuest-85), and ambient pressure drying (APD) which was carried out at laboratory oven at 70 °C during 24 h, until a constant weight of the dried products was achieved. Samples obtained by FD are denoted: A-poly-OH, A-30, A-39, A-56, A-rGO, and by APD: X-poly-OH, X-30, X-39, X-56, X-rGO, where 30, 39 and 56 represent rGO’s percentage content in the composites. 

### 2.3. Characterization 

#### 2.3.1. Textural Properties 

Textural properties: the specific surface area (SA), pores volume (Vp), average pore diameter (d_p_), porosity (P), and bulk density (D) were determined. 

Determination of SA of the examined samples was performed by the BET method. Low-temperature adsorption-desorption isotherms of N_2_ (−196 °C) were measured on a Micrometrics ASAP 2010, Nocross, GA, USA. 

Determination of V_p_ was performed on the basis of the measured volume of adsorbed N_2_ at a relative pressure p/p_o_ = 0.99. 

The average pore diameter was calculated using Equation (1):

(1) $$d_{p}=\frac{4\;Vp}{\mathrm{SA}}$$

The porosity of the investigated samples was calculated based on Equation (2):

(2) $$P=\frac{\;Vp}{D}\times 100$$

The bulk densities of the samples of aerogels and xerogels were calculated as a ratio of their weight and volume. The samples were cut into a parallelogram cube shape with dimensions 10 mm × 10 mm × 2 mm, whereas the xerogels samples had dimensions 4 mm × 4 mm × 1 mm and their weights were determined. 

#### 2.3.2. Morphology

The morphology of the investigated samples was examined using a scanning electron microscope (SEM, Hitachi S-4800, Hitacchi, Tokyo, Japan), at 15 kV accelerating voltage, after coating the samples with a thin layer of gold under reduced pressure. The samples were frozen in liquid nitrogen and afterward broken up to investigate the cross-section area, which was scanned at a desired magnification in a range of 2000×–50,000×.

#### 2.3.3. Thermal Degradation and Stability 

Thermogravimetric curves (TG) and differential thermogravimetric curves (DTG) of the investigated samples were recorded using a Thermo gravimetric Analyser model Q500 (TA Instruments, Newtown, PA, USA). The samples (~3 mg) were heated from 25 °C to 800 °C in open pans with a heating rate of 10 °C/min under a nitrogen atmosphere with a flow rate of 100 mL/min. 

#### 2.3.4. Mechanical Properties 

The dependencies of storage modulus (E’) and the loss modulus (E”) of the A-samples on frequency were recorded on an AR-G2 rheometer (TA Instruments) with 40 mm diameter parallel steel plate geometry at 25 °C. Frequency experiments were performed in the range of 0.01 to 100 Hz at a fixed oscillatory strain of 0.2% applied to the samples cut into a parallelogram cube shape with dimensions 10 mm × 10 mm × 2 mm in compression mode. The X-samples lost their structure during the measurements, forming a kind of composite powder material, and therefore their mechanical properties were not further investigated.

## 3. Results and Discussion 

**Figure 1:**
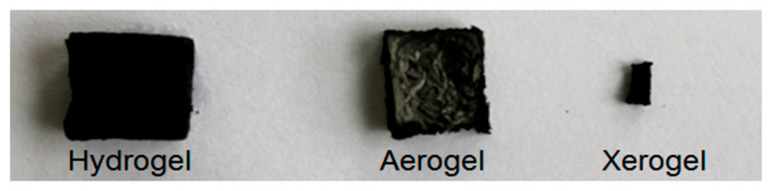
Images of the hydrogel H-56, A-56, and X-56.

Samples of poly(methylmethacrylate/butylacrylate/hydroxy-ethylmethacrylate) (poly-OH) composite hydrogels (CHG) with different rGO content, were synthesized by the described procedure and dried by the FD and APD methods. Figure 1 shows images of a representative sample of hydrogel CHG-56 and its derived composites A-56 and X-56. 

As can be seen from Figure 1, FD drying leads to the formation of A-composite samples that almost fully retain their volume with respect to the starting hydrogel sample, whereas APD drying causes a substantial decrease in the volume of obtained X-composite sample. The textural properties of the synthesized samples were investigated. The basic textural properties: D, SA, V_p_, d_p,_ and P, of the samples dried by APD are given in Table 1. 

The X-composite samples and X-rGO shows moderate D = 450–930 mg/dm^3^ and low P = 31–96%. The increase in the weight fraction of rGO in the composite leads to an increase in the values of D, SA, V_p_, d_p,_ and P. The D value of X-rGO is lower than those of the X-composites, while the values of SA, V_p_, d_p,_ and P are higher than the corresponding values of the X-composites. The determined textural properties of X-rGO and the X-composite samples with different weight fractions of rGO indicate that APD leads to structural changes in the hydrogel, which are demonstrated by the collapse of the hydrogel network due to the action of capillary forces [42]. The increase in D and P in the X-composites with the increase in weight fraction of rGO can be attributed to the number of contacts between fragments that were created due to the collapse of the network structure under the action of capillary forces. The increase in SA, V_p,_ d_p_, and P with the increase in the weight fraction of rGO indicates an increase in the relative number of fragments containing rGO sheets in relation to the samples that contain rGO sheets with bonded polymer particles. As a consequence, SA, V_p_, d_p_, and P increase [43,44,45]. 

The textural properties of samples dried by FD are shown in Table 2.

Conversely, FD of hydrogels of both rGO and composites with different weight fractions of rGO leads to the formation of samples with high P = 2000–8800% and low D = 25–53 mg/cm^3^ (aerogels). The high porosity and low D of A-rGO and A-composite samples indicate that there is no distortion and shrinking of the network structure, as well as that there is no change in the volume and structure of the porous system when they were dried by FD. The increase in the weight fraction of rGO in the CHG leads to an increase in the value of D and P, and a decrease in the value of SA, V_p_, and d_p_, of the A-composite samples. The values of SA and V_p_, d_p_, and P of A-rGO are higher than the corresponding values of the A-composites, while, on the contrary, the D value of A-rGO is lower than the D of all A-composite samples. The values of SA of A-rGO composites are much lower than SA of single graphene sheet (SA = 2640 m^2^/g) [39], most probably due to the layering or partial overlapping of flexible graphene sheets created during the self-assembly process to form a 3D network with higher density. 

As in the case of APD, there is an increase in D of the A-composites with an increase in rGO, indicating: (a) the existence of covalent interactions between polymer particles and rGO-sheets and the formation of rGO-sheets with a bound polymer; (b) mutual cross-linking between chains of rGO sheets and chains of rGO-sheets with bonded polymer particles; and (c) partial overlapping between the sheets, which leads to the formation of a 3D spatial network. Since GO is a multifunctional crosslinker, its connection with chains in the system depends on the relative contribution of GO and rGO in the reaction mixture. Therefore, with a change in the weight fraction of rGO in the composite its spatial structure changes. As the weight fraction of GO in the composite increases, the degree of cross-linking of the chains increases, which results in SA, V_p_, d_p_, and P decreasing, whereas D increases. 

**Figure 2:**
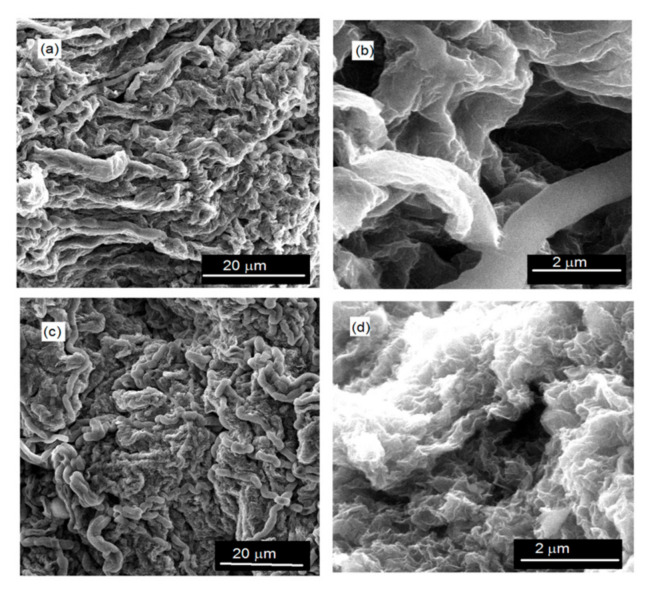
SEM images under different magnifications: (**a**) and (**b**) X-rGO; (**c**) and (**d**) X-56.

With the aim of determining the effects of the weight fraction of rGO and the drying method on the morphological properties of the synthesized composites and rGO samples, their SEM images were taken. In Figure 2, as an example, SEM images of X -rGO, and X-56 under different magnifications obtained by APD are shown. 

**Figure 3:**
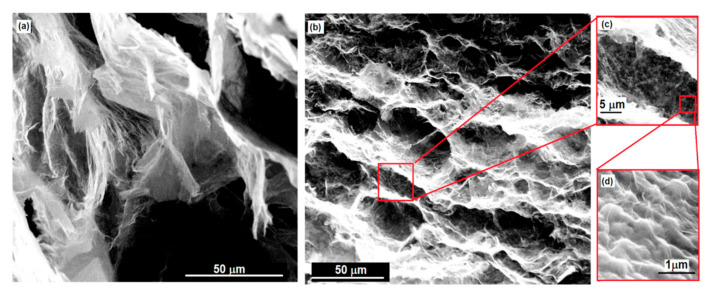
SEM images of: (**a**) A-rGO; (**b**–**d**)A-56 under different magnifications.

The presence of polymer particles, the chains of rGO sheets, and formed pores are not observed in the SEM images. The X-rGO and X-composite samples show a compact and non-porous appearance with twisted fibrous morphology. The complex, twisted fibrous morphology of the X- samples is most likely created by the mutual connection and twisting of fragments that have been formed by the collapse of the network of partially dried hydrogels. In Figure 3, as an example, SEM images of: (a) A-rGO and (b) A-56 under different magnifications are shown. 

Figure 3a,b clearly show that samples A-56 and A-rGO are highly porous. The porosity of the composite and A-rGO is most likely created by interconnection by partial overlapping of flexible rGO sheets with flexible rGO sheets with covalently bonded polymer. The formed pores are hexagonal in shape and thin-walled, with macro-porous dimensions (12–50 microns).

**Figure 4:**
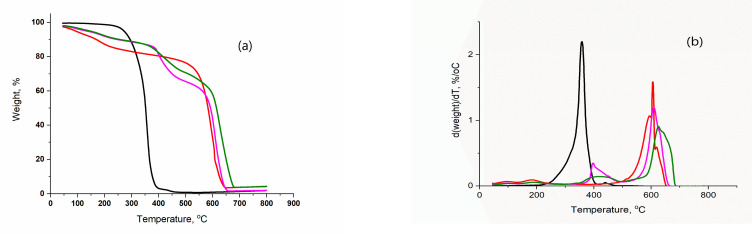
TG (**a**) and DTG (**b**) curves of the X- samples: (^_^) X-poly(OH); (**^_^**) X-rGO; (**^_^**)X-56; (**^_^**)X-39; (^_^)X-30.

In order to determine the effects of the rGO weight fraction in the X- and A-composites and the applied method of drying on their thermal stability (TS) of the obtained samples, the non-isothermal TG and DTG curves of the synthesized samples were recorded and analyzed. The TG and DTG curves of the investigated X-samples are shown in Figure 4.

The TG curves of X-composites have a complex shape. On the DTG curves of those samples, three well-separated peaks are clearly visible, indicating the existence of three stages of thermal degradation (TD) of the X-composite. The values of characteristic TD temperatures—initial temperature (T_i_), temperature of the peak’s maximum (T_m_), and final temperature (T_f_)—and the weight loss (Δm) for each TD stage of the X-samples are given in Table 3. 

The first TD stage of the X-composite, which begins at T_1,i_ = 50 °C and ends at T_1,f_ = 137 °C is followed by Δm_1_ = 5–8%. In the temperature interval of T_2,i_ = 343–374 °C to T_2,f_ = 490–520 °C, the second TD stage of the X-composite takes place with a characteristic Δm_2_ = 20–26%. The third TD stage of the X-composite starts at T_3,i_ = 580–520 °C and ends at T_3,f_ = 680–665 °C with Δm_3_ = 26–31%. 

An increase in the weight fraction of rGO in the X-composite does not lead to the change in the T_1,i_ and T_1,f_, values, whereas the values of other characteristic temperatures and weight losses decrease. The decrease in the characteristic temperatures of the second TD stage and third TD stage along with Δm_2_ and Δm_3_ indicates that the thermostability (TS) of the X-composites increases with an increase in the weight fraction of rGO in the composite. 

On the TG curve of X-poly-OH (Figure 4a), with increasing temperature, a unique sigmoidal change in the weight of the sample and, accordingly, a corresponding sharp peak on the DTG curve can be observed. The determined shape of the TG and DTG curves indicates that TD of poly-OH is performed in a single stage. The TD of poly-OH begins at T_2,i_ = 220 °C and ends at T_2,f_ = 410 °C, followed by Δm_2_ = 97.2%. 

In regard to the temperature interval at which the TD of the X-poly-OH takes place, it can be assumed that the second TD stage of the X-composite is most likely connected to the TD of the bound polymer for rGO. The values of the characteristic temperatures of the second TD stage of the TD-X composite are significantly higher than the corresponding values of the X-polymer, and the weight losses are lower, which designates a significant increase in the TS of the X-composite compared to poly-OH. Two well-separated peaks are noticeable on the DTG curve of X-rGO (Figure 4b), indicating the existence of two TD stages for X-rGO. The first TD stage of X-rGO takes place in the temperature interval from T_1,l_ = 50 °C to T_1,f_ = 131 °C and is followed by Δm_i_ = 8% and the second TD stage from T_3,i_ = 405 °C to T_3,f_ = 665 °C with Δm_3_ = 88%. In accordance with literature data, the first TD stage of X-rGO is most likely associated with the thermodesorption of adsorbed water on X-rGO, and the second stage with the decomposition of residual oxygen-containing functional groups on rGO (C=O; C-O) [45,46,47]. The determined values of the characteristic temperatures of TD of X rGO indicate that the first stage of the TD of X-composite is connected with the thermodesorption of water and the third with the decomposition of residual oxygen-containing functional groups on rGO. The values of T_1,i_ and T_1,f_ of the X-composite are identical to the corresponding ones of x-rGO, while the Δm of the composite is lower than that of X-rGO. On the contrary, the T_3,i_ and T_3,f_ values of X-composites are higher than those of X-rGO with a significantly lower weight loss, which indicates a higher TS of X-composite compared to X-rGO. 

**Figure 5:**
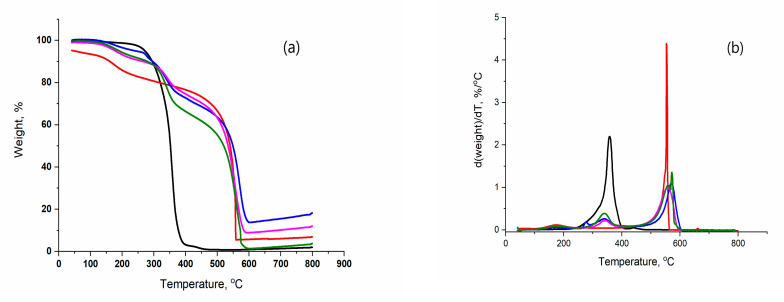
TG (**a**) and DTG (**b**) curves of the A- samples: (^_^) A-poly(OH); (**^_^**) A-rGO; (**^_^**)A-56; (**^_^**)A-39; (**^_^**)A-30.

The TG and DTG curves of the A- samples are shown in Figure 5a,b. 

The values of the characteristic temperatures: T_i,_ T_m_, and T_f_, and Δm for each TD stage of the A-samples are presented in Table 4. 

As in the case of the X-composite, the TG curves of the A composites have a complex shape. Three distinct peaks are observed on the DTG curves, which indicate the existence of three TD stages of the A-composite. The first TD stage of the A-composite begins at T_1,i_ ~104 °C and ends at T_1,f_ ~235 °C. This level of TD of the composite is followed with Δm_i_ = 5–16% and most likely, as in the case of X-composite, is associated with thermodesorption and adsorbed water on the samples.

In the temperature interval from T_2,i_ = 250293 °C to T_2,f_ = 360–388 °C, the second stage of TD of the A-composite takes place with a weight loss of Δm_2_ = 15–27%. The second stage of the TD of the A-composite is most likely connected to the TD of polymer particles bonded to the rGO sheets. The third TD stage of the composite begins at T_3,i_ = 430–478 °C and ends at T_3,f_ = 555–570 °C with Δ_m3_ = 40–66%. As in the case of the X-composite, the third TD stage of the A-composite is most likely related to the decomposition of residual oxygen-containing functional groups on rGO. Increasing the weight fraction of rGO in the composite: (a) does not lead to changes in the values of the characteristic temperatures of the thermodesorption stage; (b) causes a decrease in the value of the characteristic temperatures of TD, bound polymer, and residual oxygen groups; and (c) leads to a decrease in the value of Δm from 27% to 15%, that is, from 68% to 40%. 

The determined decrease in the characteristic temperatures and weight losses of the second TD stage and third TD stage of the A-composite with an increase in the weight fraction of rGO in the composite indicates the increased thermal stability of the composites. 

Thermal degradation of A-poly-OH begins at T_2i_ = 246 °C and ends at T_2f_ = 446 °C with sample weight loss Δm_2_ = 98%. The characteristic temperatures of the TD of the A-composite of the bound polymer for rGO are higher than the corresponding values for the A-polymer, which indicates the significantly higher thermal stability of the A-composite compared to the A-poly-OH. On the DTG curve of A-rGO samples, as well as in the case of X-rGO samples, two peaks are observed that indicate that two stages of thermal degradation of A-rGO happened. The first TD stage of A-rGO occurs in the temperature interval from T_1,i_ = 125 °C to T_1,f_ = 230 °C with Δm_1_ = 16%. The second TD stage of A-rGO, which is associated with the decomposition of residual oxygen groups containing in rGO, takes place at temperatures between T_3,i_ = 420 °C and T_3,f_ = 564 °C with a sample weight loss of Δm_3_ = 70%.

The characteristic temperatures and weight loss of the thermodesorption stage of TD A-composite are lower than the corresponding values for A-rGO. In contrast, the characteristic temperatures of T_3,i_ and T_3,f_ of the third stage of thermal degradation of the composite samples are higher than the analogous values for the A-rGO sample. The weight losses of the A-composites are significantly lower (66–44%) than those of the A-rGO samples (70%), which indicates that the A-composite samples are more thermally stable than the A-rGO samples.

Based on a comparative analysis of the established effects of the applied APD and FD on the values of the characteristic temperatures of particular TD stages of investigated samples and their weight losses during them the following can be stated. The values of the characteristic temperatures of all TD stages of the X-composites are higher than the analogous ones for the A-composite samples, whereas the values of weight losses are lower. The increase in the value of the characteristic temperatures and the decrease in the weight loss of the X-samples indicates that they are more thermostable than the A-samples. The increase in the thermal stability of the X-composites in relation to the A-composites is a consequence of their compact and non-porous nature. If the values of characteristic temperatures and weight losses of X-poly-OH and A-poly-OH are compared, it can be concluded that APD drying leads to an increase in the TS of X-poly-OH compared to A-poly-OH. The characteristic temperatures of the thermosorption TD stage of A-rG0 are higher than the characteristic temperatures of the A-composites, whereas the characteristic temperatures of the decomposition stage of residual oxygen groups of the A-composites are higher than the corresponding ones of A-rGO. This also indicates an increase in the thermal stability of rGO dried by APD. 

**Figure 6:**
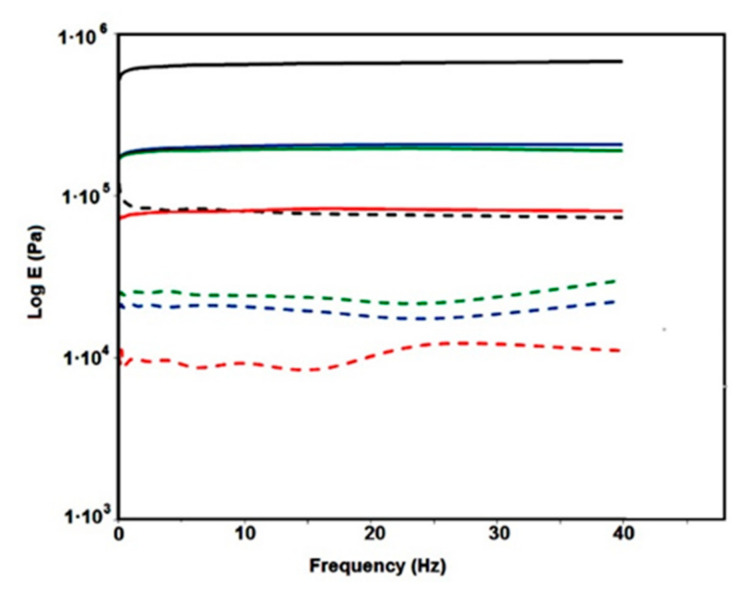
Dependence of E’ and E” on frequency for A-samples: A-rGO: (^__^)E’ and (**- -**)E′; A-56: (^__^)E’ and (**- -**)E′; A-39: (^__^)E’ and (**- -**)E′; A-30: (^__^)E’ and (**- -**)E′.

The effect of the rGO weight fraction on the mechanical properties of A-poly-OH, A-composites, and A-rGO was examined by the DMA method. Figure 6 shows the dependence of the storage modulus (E’) and the loss modulus (E”) on frequency. 

Based on the results shown in Figure 6, the following can be concluded. The values of E’ and E” of the investigated A-samples are almost independent of frequency and the dependencies of E’ and E” on frequency are parallel. The values of E’ are significantly higher than the E” for all A-composite samples. The values of E’ and E” at each frequency for A-rGO are higher than the corresponding values for each of the examined A-composites. The values of E’ and E” of the A-composites increase with the increase in the weight fraction of rGO. The independence of E’ and E” of the frequency and their mutual parallelism confirms the existence of the aerogel structure of the A-composite samples and A-rGO. The significantly higher value of E’ than E” both in the case of composites and pure rGO, at all applied frequencies, indicates the dominant influence of elastic forces on the mechanical properties of the composites and A-rGO. The increase in the E’ with an increase in the weight fraction of rGO in the A-composite leads to an increase in the mechanical strength of the composite. 

With the aim of finding an explanation for the increase in the E’ and the mechanical strength of the composite samples with an increase in the rGO weight fraction, the values of the density of crosslinking (q_c_) and the distance between two points of crosslinking (d_c_) were calculated based on the dependence of E’ on frequency [48]:

(3) $$q_{c}=\frac{E_{p}^{’}}{RT}$$

where $E_{p}^{’}$ is storage modulus on the threshold of rubber-like behavior; R is universal gas constant; and T is temperature.

(4) $$q_{c}={\left[\frac{1}{q_{c}N}\right]}^{1/3}$$

where N is Avogadro’s number. 

The effect of the weight fraction of rGO on q_c_ and d_c_ is shown in Table 5. 

The values of q_c_ increase with the increase in the concentration of rGO in the A-composite from 35 mol/m^3^ to 222 mol/m^3^, whereas the d_c_ decreases from 3.62 nm to 1.93 m. The value of q_c_ of rGO is higher than the q_c_ value for any of the A-composites, whereas the d_c_ value is lower than the values for investigated A-composites. 

By analyzing the effect of the weight fraction of rGO on the value of qc and dc of the composites, it can be concluded that the values of both q_c_ and d_c_ change linearly with the increasing weight fraction of rGO according to the equations:

(5) $$q_{c}=5.42\;w_{rGO}-8.17\;R^{2}=0.999$$

(6) $$d_{c}=3.50-0.026w_{rGO}\;R^{2}=0.985$$

where $w_{rGO}$ is the weight fraction of rGO. 

The linear dependence of qc and dc on the weight fraction of GO clearly implies that the cross-linking of the A-rGO aerogel is achieved by the mutual connection of graphene sheets in rGO, while in the case of A-composites, there is a connection of the rGO sheet with bounded polymers particles. Due to this connection, the q_c_ increases and the d_c_ decrease with the increasing weight fraction of rGO in the composite.

## 4. Conclusions

Samples of composite hydrogels with different weight fractions of rGO (0–60%) were synthesized by the method of coupled thermally induced self-assembly of GO platelets within a polymer matrix and in situ chemical reduction of GO. The effect of the weight fraction of rGO in combination with the drying method on textural properties (D, SA, V_p_, d_p_, and P), morphological characteristics, thermal stability, and rheological properties (E’ and E’’) was determined. Based on the obtained results, it is possible to draw the following conclusions. Drying composite hydrogels using the APD method leads to the formation of non-porous powders—xerogels with: (a) Moderate D = 540–930 mg/cm^3^; (b) low SA (22–42 m^2^/g), V_p_ (0.017–0.042 cm^3^/g), and P (31–45%); (c) characteristic twisted fibrous structure; (d) significant TS. Increasing the weight fraction of rGO in xerogels causes an increase in the D, SA, V_p_, d_p_, P, and TS of the xerogels. Drying of composite hydrogels by the FD method results in the formation of highly porous aerogel samples with high SA (660–920 m3/g), V_p_ (1.05–2.10 cm^3^/g), P (2000–7500%), and D (28–53 mg/cm^3^). Increasing the weight fraction of rGO in the A-composites leads to an increase in D and q_c_ and a decrease in SA, V_p_, P, and d_c_. The compact non-porous xerogels are a consequence of the collapse of the hydrogel network under the action of capillary forces during the drying of the CHG. Thermal degradation of the poly-OH occurs through a single stage, of A-rGO and X-rGO through two TD stages, whereas in the case of the A- and X-composites, three TD stages are observed (dehydration, decomposition of residual oxygen functional group, and polymer chain degradation). The thermal stabilities of the X-composites and X-rGO are higher than those of A-composites and A-rGO. The increase in D and TS, E’ and E’’, that is, the decrease in the values of SA, Vp, P, and d_c_ of the aerogels with an increase in the weight fraction of rGO is a consequence of the increase in q_c_.

## Figures and Tables

**Table 1:** Textural properties: D, SA, V_p_, d_p_, and P, of samples dried by APD.

Sample	D (mg/cm^3^)	SA (m^2^/g)	V_p_ (cm^3^/g)	d_p_ (nm)	P (%)
X-30	540	22	0.017	3.1	31
X-39	820	23	0.020	3.5	24
X-56	930	44	0.042	3.7	45
X-rGO	450	52	0.050	3.8	96

**Table 2:** Textural properties: D, SA, V_p_, d_p_, and P, of samples dried by FD.

Sample	D (mg/cm^3^)	SA (m^2^/g)	V_p_ (cm^3^/g)	d_p_ (nm)	P (%)
A-30	28	960	2.10	8.8	7500
A-39	35	880	1.80	8.1	5100
A-56	53	660	1.05	6.3	2000
A-RGO	25	1000	2.2	8.9	8800

**Table 3:** Characteristic temperatures and weight losses for each TD stage for X-samples.

	X-poly-OH	X-30	X-39	X-56	X-rGO
T_1,i_ (°C)	-	50	50	50	50
T_1,m_ (°C)	-	198	96	95	95
T_1,f_ (°C)	-	137	136	135	131
Δm_1_ (%)	-	5	5	5	8
T_2,i_ (°C)	220	374	359	343	-
T_2,m_ (°C)	350	440	420	400	-
T_2,f_ (°C)	410	520	500	490	-
Δm_2_ (%)	97.2	20	26	20	-
T_3,i_ (°C)	-	580	540	520	405
T_3,m_ (°C)	-	640	610	568	606
T_3,f_ (°C)	-	680	675	670	665
Δm_3_ (%)	-	31	29	26	88

T_i_,_j_ is temperature of a particular stage of TD where: i = 1, 2, 3 refer to TD stage and j = i, m, f refer to initial, peak’s maximum, and final T, and Δm_i_ is weight loss of particular stage of TD (i = 1, 2, 3).

**Table 4:** Characteristic temperatures and weight losses for each TD stage for A-samples.

	A-poly-OH	A-30	A-39	A-56	A-rGO
T_1,i_ (°C)	-	104	105	104	125
T_1,m_ (°C)	-	168	166	168	177
T_1,f_ (°C)	-	232	236	235	230
Δm_1_ (%)	-	7	6	5	16
T_2,i_ (°C)	246	293	280	250	-
T_2,m_ (°C)	357	350	340	295	-
T_2,f_ (°C)	446	388	380	360	-
Δm_2_ (%)	98	27	18	15	-
T_3,i_ (°C)	-	478	450	433	420
T_3,m_ (°C)	-	570			
T_3,f_ (°C)	-	628	609	590	564
Δm_3_ (%)	-	66	60	40	70

T_i_,_j_ is temperature of a particular stage of TD where: I = 1, 2, 3 refer to TD stage and j = i, m, f refer to initial, peak’s maximum, and final T, and Δm_i_ is weight loss of particular stage of TD (i = 1, 2, 3).

**Table 5:** Effect of weight fraction of rGO on q_c_ and d_c_.

Sample	E’, (kPa)	q_c,_ (mol/m^3^)	d_c,_ (nm)
Poly-OH	86.7	35.0	3.62
A-30	200	80.8	2.74
A-39	323	130.5	2.33
A-56	550	222.1	1.95
A-rGO	567	228.8	1.93
